# Glycemic Threshold as an Alternative Method to Identify the Anaerobic Threshold in Patients With Type 2 Diabetes

**DOI:** 10.3389/fphys.2018.01609

**Published:** 2018-11-13

**Authors:** Rodrigo S. Delevatti, Ana Carolina Kanitz, Cristine L. Alberton, Elisa Corrêa Marson, Patricia Dias Pantoja, Carolina DertzbocherFeil Pinho, Salime Chedid Lisboa, Luiz Fernando M. Kruel

**Affiliations:** ^1^Sports Center, Department of Physical Education, Universidade Federal de Santa Catarina, Florianópolis, Brazil; ^2^Department of Physical Education, Universidade Federal de Uberlândia, Uberlândia, Brazil; ^3^Department of Physical Education, Universidade Federal de Pelotas, Pelotas, Brazil; ^4^Exercise Research Laboratory, Department of Physical Education, Universidade Federal do Rio Grande do Sul, Porto Alegre, Brazil

**Keywords:** ventilatory threshold, second ventilatory threshold, exercise, blood glucose, diabetes mellitus

## Abstract

**Purpose:** To analyze the agreement between the velocity, heart rate, and oxygen uptake values corresponding to second ventilatory threshold and glycemic threshold in patients with type 2 diabetes.

**Methods:** Twenty-four untrained patients (55.1 ± 8.9 years) were evaluated. Three different parameters of training intensity corresponding to anaerobic threshold, one mechanical (velocity) and two physiological (heart rate and oxygen uptake) parameters, were identified by a classical method (second ventilatory threshold) and by an alternative method (glycemic threshold). To determine the threshold values, patients performed an incremental treadmill test, with an initial velocity of 3 km.h^-1^ for 3 min, that was then increased by 1 km.h^-1^ every 2 min. Comparisons between mean values and the degree of agreement between second ventilatory threshold and glycemic threshold were analyzed using the paired *t*-test and Bland-Altman test, respectively.

**Results:** All patients performed the tests appropriately, and no adverse effects were recorded. Patients demonstrated similar mean velocity (*p* = 0.25), heart rate (*p* = 0.97) and oxygen uptake (*p* = 0.71) between the ventilatory threshold (6.4 ± 0.6 km.h^-1^, 130.1 ± 18.7 bpm, 15.2 ± 3.5 ml.kg.min^-1^) and the glycemic threshold (6.2 ± 0.9 km.h^-1^, 130.2 ± 12.8 bpm, 15.0 ± 3.8 ml.kg.min^-1^).

**Conclusion:** The present study indicates an agreement between the glycemic and second ventilatory methods in determination of the anaerobic threshold of patients with type 2 diabetes; and thus, either method may be used for these patients.

## Introduction

Endurance training, generally called aerobic training in the clinical context, is strongly recommended for type 2 diabetes management (Canadian Diabetes Association Clinical Practice Guidelines Expert Committee and Cheng, 2013; Colberg et al., 2016; American Diabetes Association [ADA], 2018), as it provides both short- and long-term benefits, including improved blood pressure, lipid profile, and blood glucose, among others (Yang et al., 2014; Colberg et al., 2016; Delevatti et al., 2016a; Liubaoerjijin et al., 2016). In recent years, discussion about physical training and type 2 diabetes, especially about endurance (aerobic and/or anaerobic) training has been common, aside from the type or modality of training. Currently, studies evaluating endurance training in patients with type 2 diabetes not only compare their effects with resistance and combined training effects, but also perform many comparisons between the different ways/models of endurance training (Delevatti et al., 2016a; Asano et al., 2017; Pandey et al., 2017; Santiago et al., 2018). These studies have been evaluating acute (Terada et al., 2013; Delevatti et al., 2016b; Asano et al., 2017; Santiago et al., 2018) and chronic (Li et al., 2012; Ruffino et al., 2017; Pandey et al., 2017) effects in varying outcomes, including blood glucose, blood pressure, insulin, bradykinin, HbA1c, insulin resistance, and lipid profile. In this context, interventions have contributed to a better understanding about the role of the aerobic training variables (i.e., intensity, duration) on type 2 diabetes control.

Comparisons between different methods (i.e., continuous vs. interval training) (Karstoft et al., 2013; Terada et al., 2013; Pandey et al., 2017; Ruffino et al., 2017; Santiago et al., 2018), weekly frequencies (Vancea et al., 2009), durations (Li et al., 2012) and intensities (Asano et al., 2017) have been carried out. However, special attention should be given to intensity, because beside some classical (Boulé et al., 2001) and recent (Liubaoerjijin et al., 2016) evidence about its importance on glycemic control, this is the variable that differs most among endurance training protocols. In interval training models, such as high intensity interval training (HIIT) or sprint interval training (SIT), which have great anaerobic contribution, intensity is the main component of the exercise dosage, with generally a short duration. On the other hand, aerobic trainings with light to moderate intensity indicate a moderate to high duration (i.e., ∼30 min five times a week or 50 min three times a week) (Colberg et al., 2016; American Diabetes Association [ADA], 2018). Therefore, the intensity at which endurance training is performed is crucial to define the other training variables, such as frequency, duration, and method. A good example is shown in the guidelines for exercise and type 2 diabetes (Colberg et al., 2010, 2016; American Diabetes Association [ADA], 2018), that define the duration (150 min, 75 min, or a combination of these) of aerobic training regarding its intensity (moderate, vigorous, or a combination of these, respectively).

Despite the importance of intensity in endurance training, most studies with aerobic training for patients with type 2 diabetes prescribe the intensity without adequate knowledge of the metabolic stress (i.e., blood lactate) in which patients exercise. This occurs due general use of the percentages of the physiological maximum parameters, including heart rate percentage (%HR_max_) and oxygen uptake percentage (%VO_2max_), also called “relative percent method” (Wolpern et al., 2015). Despite its broad recommendation, this approach does not accurately represent metabolic stress (Wolpern et al., 2015). The same point (i.e., 70% VO_2max_) can represent a moderate or high intensity for individuals with different training status-cardiorespiratory fitness. Even performing incremental tests to determine the real maximum parameters of the patients, the classical approach of intensity determination does not perfectly contemplate the biological individuality principle, as changes in anaerobic threshold in response to training without changes in the maximum condition can occur (Scharhag-Rosenberger et al., 2012; Delevatti et al., 2016a). In addition, there are differences between individuals in these parameters. In patients with type 2 diabetes, this can be confirmed by the dispersion existing in the anaerobic threshold values in relation to the maximum cardiorespiratory capacity (i.e., %VO_2peakAT_), as well as by the variance of this parameter between studies (Belli et al., 2007; Delevatti et al., 2016a; Sousa et al., 2016). Thus, individuals with the same maximum condition can have their anaerobic threshold in different percentages of maximum capacity, presenting different internal loads for the same maximum percentage.

For a better prescription of the endurance training, determining anaerobic threshold is necessary because it represents an important metabolic state, providing a more accurate understanding of the physiological response to exercise, besides being more responsive to training than the maximum parameters (Meyer et al., 2005). Previous studies (Kawaji et al., 1989; Fujita et al., 1990) have shown that anaerobic threshold is a direct, simple, and useful parameter that should be considered when prescribing the optimal exercise intensity in patients with type 2 diabetes. In these patients, the anaerobic threshold represents an intensity where plasma glucose level decreases without a substantial increase in plasma glucagon concentrations and minimizes the risk of a cardiac accident (Kawaji et al., 1989).

Different methods have been applied to obtain anaerobic threshold, and the best-known methods involve the use of blood lactate analysis and a ventilation curve. Blood lactate analysis allows the maximum load performed during exercise to be determined, in which balance between lactate production and removal occurs. This point is generally called the second lactate threshold (Binder et al., 2008). The ventilatory curve and ventilatory equivalent analyses enable the determination of the break point at which the respiratory system is unable to effectively buffer H^+^ ions, which leads to a disproportional increase in ventilation and carbon dioxide. This break point is known as the second ventilatory threshold (VT2)(Reinhard et al., 1979; Binder et al., 2008). However, the high cost of the equipment used to analyze breathing gasses makes the method very expensive, limiting its practical application (Silva et al., 2006; Azevedo et al., 2009).

A less expensive and more accessible alternative to determine anaerobic threshold can be achieved by analyzing glycemic behavior. Studies comparing blood glucose and lactate in non-diabetic individuals have been completed (Simões et al., 2003; Souza et al., 2003; Mendes et al., 2011). After inducing lactic acidosis with sprints, young individuals performed an incremental test to determine the velocity and heart rate corresponding to minimum lactate and minimum glycaemia. The authors (Souza et al., 2003) then compared these values with velocity and heart rate corresponding to lactate concentration of 4 mmol/L. No differences were found in the two parameters (velocity and heart rate) among the three methods (minimum lactate, minimum glycaemia, and 4 mmol/L concentration) of anaerobic threshold determination. In another study (Simões et al., 2003), anaerobic threshold velocity analyzed by blood glucose, blood lactate, or ventilation was similar in young men. This behavior was found in incremental tests with and without inducing previous lactic acidosis. In contrast, Mendes et al. (2011) did not find agreement between glycemic threshold (GT) and the maximal lactate steady state in young men. It is important to note that these studies were carried out with young and active individuals, with a small sample size, with lactate markers used as gold standard for comparison with GT. Thus, more studies are needed to evaluate GT in a higher sample size, with other populations, as diabetic and sedentary patients, in comparison with the VT2 method.

The main basis for GT is the action of counter-regulatory glycemic hormones, which are enhanced by exercise intensities above the anaerobic threshold. This physiological mechanism has been evaluated and discussed in high-intensity protocols in patients with type 1 diabetes (Simões et al., 1999; Bussau et al., 2006; Farinha et al., 2017), but is not associated with anaerobic threshold concepts or type 2 diabetic patients.

To determine the anaerobic threshold the practicality of measuring capillary glycaemia offers a distinct advantage over the lactate measure and the VT2 (which requires the use of a mask), because patients are usually familiar with glucometers. In addition, the analysis of GT in patients with type 2 diabetes has important implications and possible differences compared to other populations. These patients present a higher ratio of glycolytic:oxidative enzyme activities within skeletal muscle (Simoneau and Kelley, 1997), use inhibitors of hepatic glucose production (American Diabetes Association [ADA], 2018) and have glycaemia as the most important variable, which is totally associated to risks and benefits in diabetic treatment.

Nevertheless, information regarding the agreement between the GT and VT2 methods is difficult to ascertain, especially in patients with type 2 diabetes. Further, the possible representation of the same intensity by these two methods remains to be tested. We believe that for a broad application of anaerobic thresholds in type 2 diabetes management, it is necessary to present the findings in different parameters (physiological and mechanical – such as the velocity achieved during the test) and to discuss the particularities (difficulties and possibilities) involved in choosing and evaluating these variables over time. Therefore, the present study aimed to analyze the agreement among the velocity (mechanical parameter), heart rate (HR) and oxygen uptake (VO_2_) values (physiological parameters) corresponding to VT2 and GT in patients with type 2 diabetes. Our hypothesis was that all the parameters evaluated should correspond to the same threshold in both methods, leading to a good agreement between VT2 and GT methods.

## Materials and Methods

### Subjects

After approval of this study by the Research Ethics Committee of the Federal University of Rio Grande do Sul (n°: 108.997) and by the Research Ethics Committee of the Clinical Hospital of Porto Alegre (n°: 54475), 24 subjects (13 men and 11 women) with type 2 diabetes and aged > 30 years (between 37 and 71 years) provided written consent to participate in this investigation. Patients with the following conditions were excluded from the sample: uncontrolled hypertension, autonomic neuropathy, severe peripheral neuropathy, proliferative diabetic retinopathy, severe nonproliferative diabetic retinopathy, decompensated heart failure, limb amputations, chronic renal failure (Modification of Diet in Renal Disease-glomerular filtration rate < 30 mL/min) (Meara et al., 2006), or any muscle or joint impairments that prevented individuals from engaging in physical exercise. The absence of these conditions was confirmed by medical history as well as by clinical and laboratory examinations. All patients underwent electrocardiogram stress testing 6 months prior to the study.

### Experimental Procedures

Before performing the exercise tests proposed in this study, all patients underwent anthropometric measurements, fasting blood sampling, and familiarization with the exercise test.

### Anthropometry

An initial session was held to collect anthropometric data. Body mass and height measurements were obtained using an analog medical scale and a stadiometer (FILIZOLA; Sao Paulo, Brazil). Based on these values, the body mass index was calculated according to the following equation: Body mass index = mass (kg) × height (m)^-2^.

### Blood Analysis

Blood samples (4 ml) were obtained from the antecubital vein after fasting for 12–14 h. The samples were collected in tubes with EDTA and were frozen at -80°C as total blood (without centrifugation). After blood data collection, the levels of glycated hemoglobin were determined through high-performance liquid chromatography to characterize the glycemic chronic status of the patients.

### Exercise Test

Exercise tests were conducted on a previously calibrated treadmill (Inbramed, Porto Alegre, Brazil) with an initial velocity of 3 km.h^-1^ for 3 min, with a fixed incline (1%); the velocity was increased by increments of 1 km.h^-1^ every 2 min, until exhaustion. Heart rate was monitored every 10 s (Polar, Kajaani, Finland), and capillary glycemia, as well as the rate of perceived exertion, were measured in the final 20 s of each stage. An Accu-Chek–Multiclix lancet device was used to access capillary blood, and capillary glycemia was assessed using a clinical glucometer (Accu-Check Performa, Roche, São Paulo, Brazil), which assesses glycemic levels in approximately 5 s. This blood glucose monitoring system presented great accuracy, with 99.5% of values analyzed within accuracy limits that were internationally standardized (Freckmann et al., 2012). Furthermore, during the test, oxygen uptake, carbon dioxide production (VCO_2_), and ventilation (Ve) were continuously monitored using a portable gas analyzer (VO2000 Gas Analyzer, Med Graphics) with sampling frequency of one sample every three breaths. Prior to each session, the portable gas analyzer was calibrated according to the manufacturer’s instructions. The assessment was considered valid when some of the following criteria were met at the end of the test: 1) estimated maximal heart rate was reached (220 – age); 2) a respiratory exchange ratio greater than 1.15 was achieved; and 3) a rating of perceived exertion of at least 18 on the Borg scale of 6–20 (Howley et al., 1995). All tests were performed in the presence of a cardiologist, at a controlled room temperature (24–26°C).

### Criteria for Determining the VT2 and GT

**FIGURE 1:**
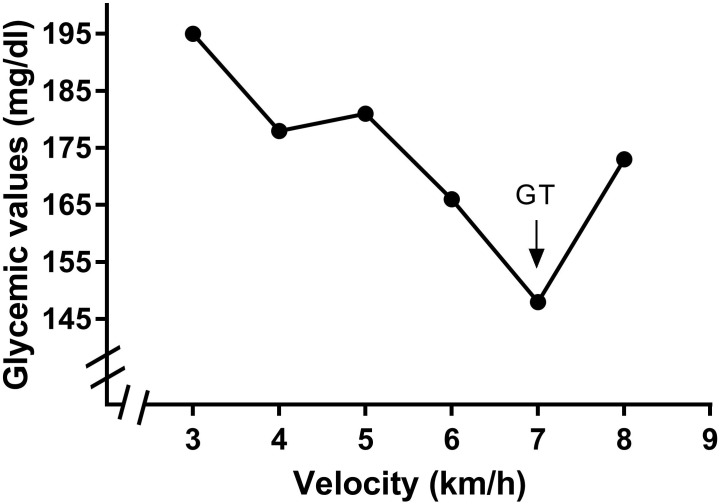
Identification of the glycemic threshold (GT) in a participant of the study. Identification was performed by lowest glycemic value found in a exercise incremental test performed in treadmill.

The VT2 was obtained by determining the second inflection point in the ventilation curve and was confirmed using the CO_2_ventilatory equivalent (Ve/VCO_2_) (Wasserman et al., 1973). The curves were analyzed by two independent, blinded, experienced exercise physiologists. The break points corresponding to the VT2 were considered valid when both analysts identified the same value. If there was no consensus, a third physiologist was recruited, and after his analysis, the break point was selected using the median of the points found by each exercise physiologist. The GT was obtained by determining the intensity corresponding to the lower glycemic level followed by an increase during test performance (Simões et al., 1999, 2003; Mendes et al., 2011). An example of the GT determination is presented in Figure 1.

Data analysis was based on the velocity, heart rate, and oxygen uptake corresponding to VT2 and GT.

### Statistical Analysis

For descriptive analysis, data are presented as mean ± standard deviation. Shapiro-Wilk’s test was used to verify the normal distribution of the data. Comparisons between the GT method and the VT2 reference method were performed using the paired *t*-test and Bland–Altman method, which evaluates the potential existence of agreement or bias. Bland–Altman analysis was used to determine means and standard deviations to evaluate differences between measurements acquired from the standard and new methods. By analyzing bias and limits of agreement, it is possible to evaluate whether the methods agree. Depending on the nature of the variable, a wide limit of agreement may represent the absence of agreement between methods. A bias close to zero represents an agreement between methods. Associations among variables also were analyzed by the Pearson product-moment correlation coefficient.

## Results

All patients performed the tests appropriately, and no adverse effects were recorded. Characterization data are presented in Table 1.

**Table 1:** Patients characteristics.

Age (years)	55.1 ± 8.9
Durationof DM2 (years)	9.0 ± 6.4
HbA1c (% - mmol/mol)	8.0(64) ± 2.6(13)
Height (cm)	166.0 ± 9.0
Bodymass (kg)	86.9 ± 16.3
Body mass index (kg.m^-2^)	31.1 ± 4.7
Medication	
Metformin	22
Sulphonylurea	3
DPP-4-inhibitors	2
Insulin	4

*DPP-4, dipeptidyl peptidase-4. Values of age, duration of DM2 and anthropometric measures are expressed as the mean ± SD; Values of medication are expressed by n*.

Mean velocity, HR, and VO_2_ corresponding to GT and VT2 are presented in Table 2. According to the paired *t*-test, there were no significant differences between the GT and VT2 methods (Mean velocity, *p* = 0.25; HR, *p* = 0.97; VO_2_, *p* = 0.71).

**Table 2:** Velocity, heart rate and oxygen uptake corresponding to second ventilatory threshold and to glycemic threshold.

	VT2	GT
Velocity (km.h^-1^)	6.4 ± 0.6	6.2 ± 0.9
HR (bpm)	130.1 ± 18.7	130.2 ± 12.8
VO_2_ (ml.kg.min^-1^)	15.2 ± 3.5	15.0 ± 3.8

*VT2, second ventilatory threshold; GT, glycemic threshold; HR, heart rate; VO_2_, oxygen uptake. Values are expressed as the mean ± SD*.

**FIGURE 2:**
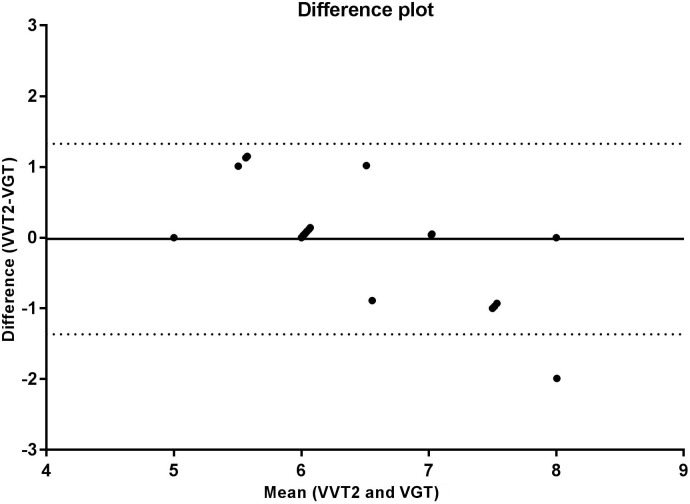
Bland Altman test for velocities corresponding to the second ventilatory threshold (VT2) and glycemic threshold (GT), in sedentary patients with type 2 diabetes. The solid line near zero represents bias (–0.208), and the dotted lines represent the limits of agreement (–1.940 – 1.524).

**FIGURE 3:**
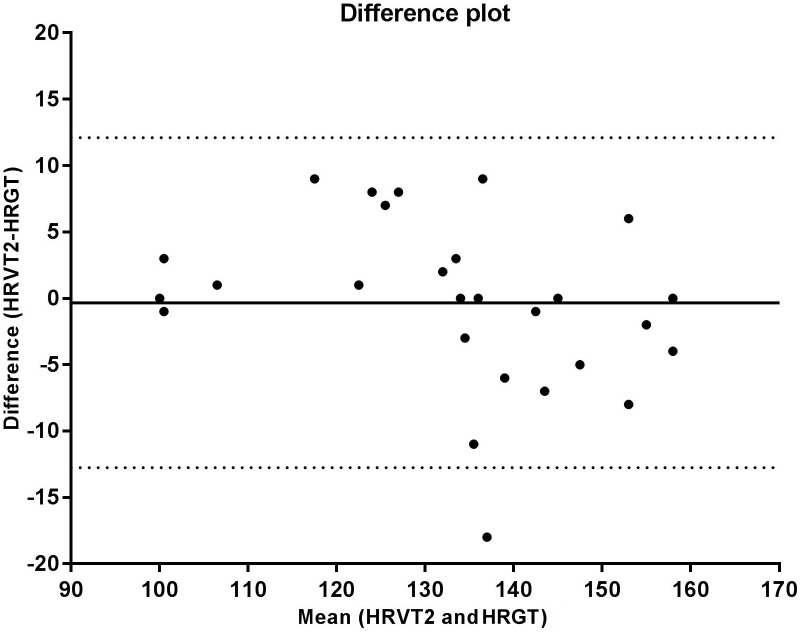
Bland Altman test for heart rate values corresponding to the second ventilatory threshold (VT2) and glycemic threshold (GT), in sedentary patients with type 2 diabetes. The solid line near zero represents bias (–0.090), and the dotted lines represent the limits of agreement (–29.71 – 29.53).

**FIGURE 4:**
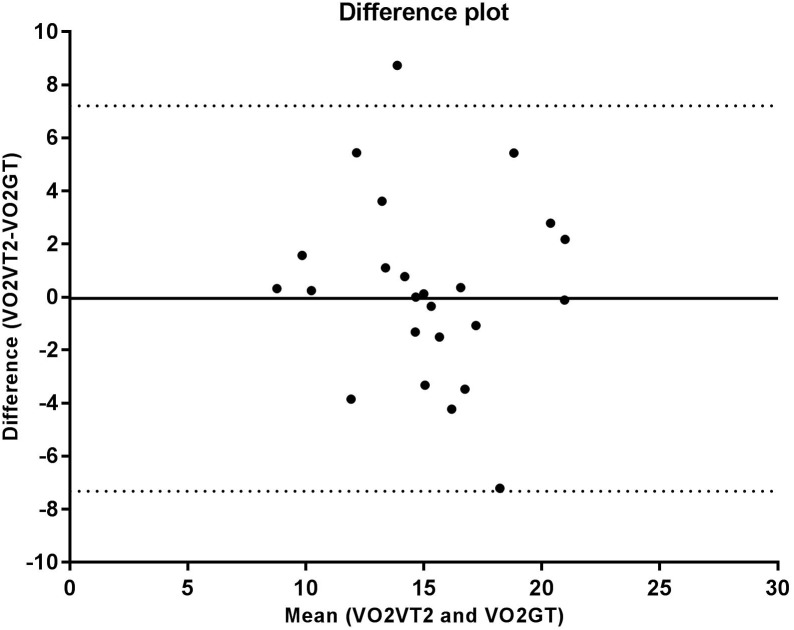
Bland Altman test for oxygen uptake (VO_2_) values corresponding to the second ventilatory threshold (VT2) and glycemic threshold (GT), in sedentary patients with type 2 diabetes. The solid line near zero represents bias (–0.050), and the dotted lines represent the limits of agreement (–7.32 – 7.21).

The correlation analysis between the GT and VT2 showed a weak and non-significant correlation for velocity (*r* = 0.348, *p* = 0.095), a moderate and significant correlation for VO_2_ (*r* = 0.569, *p* = 0.004), and a strong and significant correlation for HR (*r* = 0.714, *p* < 0.001). According to the Bland-Altman analysis, the limits of agreement represent a small amplitude range, especially for mean velocity, with the bias close to zero. Hence, an agreement is assumed between the two methods used to determine anaerobic threshold. This agreement is presented for velocity (Figure 2), HR (Figure 3), and VO_2_ (Figure 4).

## Discussion

Results from the present study indicate an agreement between the glycemic and ventilatory methods in untrained patients with type 2 diabetes. This agreement was found in all parameters evaluated (velocity, HR, and VO_2_), confirming our hypothesis.

Although not widespread in clinical practice, glycemic behavior during maximal exercise tests has been the target of several investigations in exercise science (Simões et al., 1999, 2003; Mendes et al., 2011). During an incremental test performed on physically active subjects who had lactic hyperacidosis, the velocity corresponding to the lower glycemic value was not significantly different from the lactate minimum velocity (Souza et al., 2003). The association between blood glucose and blood lactate has also been investigated in endurance runners, and no differences were found between the GT and lactate threshold after incremental tests performed on a track (Simões et al., 1999). Even though these studies enable us to better understand the lactate threshold phenomenon, they were performed on an active and healthy population. Moreover, studies usually compared GT to the lactate threshold, but have not compared GT with VT2, which hinders further discussion on the results of the present study.

Clinically, our results have great relevance, indicating the agreement between different methods of determining the anaerobic threshold in patients with type 2 diabetes. This finding has particular importance in patients with a pathologic condition, which is associated with mitochondrial dysfunction (Kelley et al., 2002), a higher ratio of glycolytic:oxidative enzyme activities within skeletal muscle (Simoneau and Kelley, 1997), higher lactate levels (Crawford et al., 2010), and the use of inhibitors of hepatic glucose production (American Diabetes Association [ADA], 2018), which may influence the determination of the anaerobic threshold.

In addition, scientific literature already supports the benefits of prescribing exercises with intensities corresponding to the anaerobic threshold in this population (Belli et al., 2007, 2011; Brun et al., 2008; Delevatti et al., 2015). Other important finding of the literature that favors use of the anaerobic threshold and not the maximum relative parameters is the cardiorespiratory fitness responsivity to these methods. In a randomized clinical trial performed by Wolpern et al. (2015), 100% (12 of 12 individuals) of responsivity on VO_2max_ in sedentary individuals trained by progressive ventilatory-thresholds model was found, while only 41.7% (5 of 12 individuals) of responsivity was found in individuals trained by percentages of the reserve heart rate. Although the study of Wolpern et al. (2015) was with a non-diabetic population, the improvement of cardiorespiratory fitness is vital for patients with type 2 diabetes (Johannsen et al., 2013) because it is strongly associated with glycemic control (Lidegaard et al., 2015). This reinforces the use of the anaerobic threshold in the population of the present study.

The agreement found in the present study allows for further exploration of a low-cost method that can be applied easily in different therapeutic settings and is accessible to diabetic patients that are already familiar with blood glucose measurement. In contrast, the ventilatory method is expensive and uncomfortable, as it requires a mask for the collection of respiratory gasses.

Besides the possible application of a clinical glucometer to determine the anaerobic threshold in diabetic patients, it is important that both methods show similar results during treadmill exercise. Indeed, studies (Oliveira et al., 2012; Qiu et al., 2014) have demonstrated that walking/running are the most used and investigated modality of aerobic training in type 2 diabetes management. In addition, in prescribed walking or running training, more muscle mass is recruited than that in other forms of aerobic training, such as the ergocycle. This improves body composition and glycemic control (Belli et al., 2007).

For practical implications of this study, exercise professionals need to choose one of the three parameters evaluated (velocity, HR and VO_2_) and understand its particularities. Velocity is a mechanical parameter, easily controlled in treadmills or with electronic devices. However, velocity is a parameter of external load and can suffer interference from many factors, such as time, previous exercise, fatigue, and sleep alterations (Delevatti et al., 2015). To use this parameter in long-term periodization, it is important to reassess it frequently, because with cardiorespiratory adaptations to training, higher external loads should be necessary, like velocity for a same internal load. Besides, it is necessary caution in choose of the GT velocity, for even presenting good agreement with VT2 velocity, did not found significant correlation between these variables. Regarding the use of HR, a physiological parameter, there is a slower interference of the other factors, such as previous exercise, fatigue, and sleep. This parameter may be used to modulate the training intensity in accordance to the current situation of the patient, with adjustments in the intensity, based on previous effort or rest (Delevatti et al., 2015). Even so, revaluations are necessary, because with cardiorespiratory adaptations, the anaerobic threshold should be found in higher internal loads, represented by higher HR and VO_2._ Even though the VO_2_ is not directly used in the training prescription, it is used to estimate energy expenditure in exercise. For this, our results also show that GT can lead to the same energy expenditure when using the same session duration and intensity, as determined by VT2.

Investigating the association of the GT and VT2 in patients with type2 in only one exercise modality (walking/running) and one training status (untrained) are limitations of the present study. However, the strengths of the study include the use of an alternative method to identify the anaerobic threshold in a highly prevalent disease, the discussion of the advantages and disadvantages in the application of the GT and of the VT2, and the emphasis on the importance of increasing prescription and control of training intensity regarding the anaerobic threshold.

## Conclusion

In conclusion, this study found an agreement between the GT and VT2 methods in untrained patients with type 2 diabetes, suggesting that the GT should be the target of future investigations for strengthening its use in clinical practice. It is important to increase the prescription and control of training intensity based on the anaerobic threshold, because knowledge of this metabolic state point increases the safety and effectiveness of exercise interventions for controlling type 2 diabetes.

## Author Contributions

RD, AK, and LK designed the study, obtained the funding, researched and analyzed the data, wrote the manuscript, contributed to the discussion, and reviewed and edited the manuscript. AK, CA, and PP researched and analyzed the data, contributed to the discussion, reviewed and edited the manuscript. EM, CP, and SL participated in the collection and tabulation of the data. All authors approved the final version of manuscript.

## Conflict of Interest Statement

The authors declare that the research was conducted in the absence of any commercial or financial relationships that could be construed as a potential conflict of interest.
